# The Orthopædic Department

**Published:** 1914-05-02

**Authors:** 


### THE ORTHOPÆDIC DEPARTMENT.*

(Contributions to this Section are Invited.)

### VI. The Plaster Room.

One of the most important components of the orthopædic unit is the plaster room, wherein all those modifications of plaster work that constitute such an important part of modern orthopædic work are carried out. During the last five years the utility of such manipulative work has become increasingly recognised in this country; it has long ago been realised abroad that plaster work is an indispensable help to the orthopædist, and a part of his work which needs very careful study and practice. Much of the treatment which must be applied in the unit belongs to the class that is popularly, though perhaps erroneously, styled "bloodless surgery."

### Plaster Rooms Abroad.

The plaster room should be a part of the out-patient unit of the main unit, since much of the work can easily be applied to out-patients who are treated as "ambulants"; while in-patients, needing more care and closer supervision, can easily be brought down to the out-patient department at such times as the room is not occupied by regular out-patients. A moderate-sized room serves best. In Continental clinics, where several patients are treated simultaneously, the plaster room is often one of the largest in the unit. This is, perhaps, a mistake, except in cases where the staff is large and where there is a great pressure of work. Even then it is better to have two or more rooms, at the risk of duplicating the machinery. Plaster work needs careful and methodical adjustment, and should never be hurriedly done. In some cases the patient has to be anæsthetised—as in a case of congenital dislocation of the hip joint—and the actual work of adjusting the plaster demands the attention of at least three assistants. A comparatively large staff must, therefore, be available to supervise simultaneous work on three or more patients, and under such conditions the attention of the workers is apt to be distracted when several operations are proceeding simultaneously in the same room.

### Points in Construction.

The room should be well lighted, with light walls and ceiling, impervious flooring, preferably tiles or terrazzo, though a thick linoleum floor on a cement foundation will serve if it is carefully looked after and methodically cleaned. Adequate and ample sink accommodation, with hot and cold water, should be provided. Here it is desirable to lay stress on the form and position of the sinks. Two at least of these should be placed low down close to the floor, not higher than two feet above the floor in fact, and these two special sinks should be at least eighteen inches deep and three feet long. They serve as patients' limb baths when it is desired to soften old plasters by immersion in water for some time. To take off an old plaster is often a difficult matter that needs time and patience; if the patient is directed to immerse the whole limb, upper or lower, in the water-bath in the sink—if he or she is capable of such self-help—much time is saved to the assistants, as the patients themselves may then easily remove the upper layers of the plaster, or even the whole. The washing basins should be roomy enough to accommodate the surgeon's forearm with ease—another trifling detail that is often overlooked—and hot and cold water, with a mechanical arrangement to permit of mixing the two streams, should be provided.

### Instrument-Makers' Special Cupboards.

In the centre of the room, well away from the other furniture, should be the work-table, of strong, heavy wood, preferably not on castors, but on broad, square legs, so that it cannot easily shift its position; to this table can easily be affixed the various leg and arm rests which may be required on occasion; these should be kept handy in a special apparatus cupboard. Another cupboard will be required for splints, a third for bandages and strengthening material, and a fourth for plaster. Instrument-makers list special cupboards, usually at a far higher price than they are worth. As a matter of fact any expert carpenter can easily manufacture such cupboards to pattern from half-inch bass wood, painted outside with white enamel paint. Such carpenter-made cupboards look every whit as good as the more expensive furniture in the dealers' catalogues, and serve the same purpose admirably at a far less expense. Another cupboard will be required for instruments; this is preferably an ordinary glass-shelved instrument cupboard-case. A small glass stand for the anæsthetist, with tray for bottles and drawer for instruments, must also be provided, as well as a few adjustable chairs for anæsthetist and assistants. The small metal-enamelled stools are admirably suited for this latter purpose. It is preferable not to keep the apparatus for Bier's treatment in the plaster room, but to reserve the room entirely for the use of those who are doing plaster work.

### The Plaster Itself.

A word must now be said with regard to the materials used in the work. Of these the most important is plaster of Paris, gypsum, or well-dried calcium sulphate. This is a white powder, which should be free from lumps and from commercial impurities, such as carbonates or oxides of lime. Two qualities of plaster are usually stocked. One is the finer and purer plaster used for bandages, and the other the less pure and coarser plaster used by masons and builders, which is economical for use in taking plaster casts and in strengthening plaster jackets, etc. The second quality is much cheaper, but both qualities are best stocked in small tins, air-tight and soldered down, or with air-tight, easily-opened lids. A plaster that needs

* Previous articles appeared on November 1, 15, 29 and December 13, 1913, and January 24, 1914.

baking or other preparation before it can be used, is, in the long run, less economical and more unsatisfactory than the slightly more expensive grades. Surgeons generally prefer the special brands, with which they are more familiar, especially for bandaging work, and there are many kinds listed. Personally, we have found the white "Alabaster" plaster, which is extensively used in Germany, the best for bandages, and the coarser "masons' plaster" suitable for other purposes. The great point is that the plaster should be free from lumps and grit; earthy impurities are far more inconvenient than is the presence of chemical impurities—such as the carbonates, for instance—since the latter are to some extent dissolved by the water when the plaster is made. If care is taken to keep the tins air-tight, and to prevent access of moisture to the cupboard wherein the plaster is stored, the material will always be fit for use. Tins that have stood open for some time should be emptied and the plaster used for casting, or mixed with the coarser grade.

### Methods of "Setting."

A variety of materials is used for hastening the "setting" of plaster; good plaster scarcely needs such adjuvants, and they need merely be mentioned here. Among the substances most commonly used, and of which a small stock should be kept in the plaster cupboard for use when required, are chloride of ammonium, common salt, alum, barium sulphate (used with the so-called "magnesite bandages"), chalk, fine Portland cement or silver sand, and oak or maple sawdust. Some of these are also used for stiffening and strengthening bandages made of glue or gum.

In addition to plaster, bandages of starch, glue, stearine, gum, water-glass, or certain proprietary substances, such as magnesite, are used. A supply of these materials should, therefore, be included in the stock of the plaster room. The silicates are sometimes very useful, although supports made of water-glass are unsatisfactory in comparison with pure plaster or celluloid bandages. Starch and glue bandages are convenient to work with, easily manufactured, and present many other points of advantage. The ordinary laundry starch and the best carpenters' glue are the most satisfactory; with regard to the latter the cakes should be transparent, and a proper glue-pot should be provided, with several differently sized brushes. Nearly all these bandages need strengthening if they are to last for any length of time. The chief strengthening materials used are thin strips of tin or lead, wire gauze, aluminium strips or foils, thin laths of some light wood, cardboard, felting, or artificial wood, and Portland cement. This last is an excellent strengthening material when combined with plaster and gum acacia, and is much used abroad.

### Celluloid.

Finally, we come to celluloid, which now competes, to replace plaster of Paris, in orthopædic work. It is easily worked with, is clean, light, resists acids and ordinary wear and tear better than does plaster, and splints made from it are almost impervious. It has the great disadvantage of being inflammable, but this can be overcome by mixing it with certain substances, such as aluminium chloride. It is best stored as shavings, obtainable from any celluloid works. Such shavings must be dissolved in acetone or petrol, the former being by far the easier solvent. The acetone must be stored in bottles, and the solution freshly prepared when wanted, although a stock preparation may be kept in a wide-mouthed bottle for use in emergencies.

### Preparation of Celluloid Bandages.

We shall later on give a description of the method employed in preparing celluloid bandages and splints. The basis of the plaster or celluloid bandages is in all cases some material which will "hold" the paste or solution used. Any cloth that does this will serve. For the coarser kinds of splinting a fairly heavy canvas does admirably; for the finer bandages a much thinner cloth is preferable. Butter muslin is used for nearly all the finer bandages, though some surgeons prefer a material which is slightly more resistant, and use a cotton or linen bandage. This latter, however, does not hold the plaster so well as does the finer butter muslin. Flannel and calico bandages are also required. Rolls of the bandaging material must be kept in the store cupboard, together with sewing needles and thread, a few spare pairs of ordinary scissors for cutting out, a dozen or so of ordinary hose, and, as the basis for plaster jackets, variously sized undervests of thin tricot or cotton.

